# Editorial: Lymphatic system: organ specific functions in health and disease, volume II

**DOI:** 10.3389/fcell.2025.1659266

**Published:** 2025-07-29

**Authors:** Zoltán Jakus, Young-Kwon Hong, Tsutomu Kume

**Affiliations:** ^1^Department of Physiology, Semmelweis University, Budapest, Hungary; ^2^Keck School of Medicine, University of Southern California, Los Angeles, CA, United States; ^3^ Northwestern University Feinberg School of Medicine, Chicago, IL, United States

**Keywords:** lymphatic, lymphangiogenesis, edema, lymphangiocrine factors, lymphatic abnormalities

## Introduction

The lymphatic system plays central roles in the maintenance of fluid balance, clearance of macromolecules, fat absorption, and immune surveillance. Lymphatic functions vary notably across organs and physiological contexts and play important roles in numerous pathological conditions (Janardhan et al., 2023; Mehrara et al., 2023). Despite recent progress in understanding these functions, the cellular and molecular mechanisms that govern the formation and regulation of lymphatic vascular heterogeneity in various organs and tissues remain incompletely defined. This Research Topic brought together a Research Topic of four original research articles and five review articles from diverse disciplines - developmental biology, lymphatic malformations, inflammation, pathophysiology, bioengineering, and potential treatments - to deepen our understanding of organ-specific lymphatic functions in health and disease.

## Lymphatic morphogenesis and organ-specific vasculature

Vascular endothelial growth factor receptor 3 (VEGFR3) signaling in lymphatic endothelial cells (Kuonqui et al.). This is a comprehensive review detailing VEGFR3 pathway regulation in lymphatic development and function. It outlines key mediators downstream of the VEGFC/VEGFR3 signaling pathway in lymphatic endothelial cells (LECs) and their functional roles in lymphatic vessel physiology and discusses the regulation of overall VEGFR3 activity in lymphatic vessels.

Lymphangiocrine signaling pathways in the heart and intestine (Kurup et al.). Recent evidence indicates that LECs secrete paracrine (lymphoangiocrine) molecules that are specific to organ tissues. This review article provides a comprehensive overview of the crosstalk between LECs and adjacent cells to augment tissue regeneration in cardiac and intestinal disease. The roles of lymphangiocrine factors, including Reelin, Apelin, Adenomedulin, and R-Spondin 3, in the heart and intestine in tissue maintenance and self-renewal following injury are discussed.

Lymphatic valve formation and function during development (Davis et al.). This original research article is aimed at investigating the developmental progression of functional lymphatic valve leaflets that prevent backflow to maintain a unidirectional flow. Lymphatic valve development includes four main stages (stages 1–4) of morphogenic dynamics. *Ex vivo* tests of valve function using isolated murine mesenteric collecting lymphatic vessels reveal that valves become functional, preventing backflow between stages 3 and 4 of development.

Regulation of KRAS/MAPK signaling in lymphatic vessel development (Fernandes et al.). Somatic activating mutations in *KRAS* cause complex lymphatic anomalies (CLAs). However, its pathogenesis remains largely unknown. This original research article demonstrates that hyperactive KRAS signaling caused by the KRAS mutation (p.G12D) results in enlarged lymphatic vessels in mouse embryos. This mutation induces cell spindling, proliferation, and migration *in vitro*. Importantly, this study also reveals the therapeutic potential of targeting hyperactive KRAS signaling in lymphatic anomalies associated with somatic KRAS mutations.

These articles provide insights into the various functions of lymphatic vessels in different organs and tissues and how the extracellular signaling pathways, ranging from VEGFC/VEGFR3 to lymphangiocrine factors, contribute to maintaining proper lymphatic function and tissue homeostasis.

## Pathophysiology and disease mechanisms

Secondary lymphedema molecular pathology (Lee and Kim). This review article provides a comprehensive overview of the pathophysiology of secondary lymphedema, a condition that arises due to injury or obstruction of the lymphatic system, and most commonly and globally, filariasis. It highlights three consequences in lymphedema: (1) chronic inflammation-mediated lymphangiogenesis, which is dependent on the VEGF-C/VEGFR axis, (2) adipocyte hypertrophy and adipose tissue deposition, and (3) tissue fibrosis. Despite surgical treatments of reconstructing the lymphatic system to facilitate lymphatic fluid drainage, there is a limitation of their effectiveness in treating already damaged lymphatic vessels.

Lung lymphatics in chronic obstructive pulmonary disease (COPD) (Trivedi et al.). Using *in vitro* and *in vivo* models, this original research article reveals inflammatory and fibrinogenic alterations in pulmonary lymphatics within cigarette smoke-augmented COPD models. Immunohistochemistry of human lung tissue also shows increased expression of inflammatory markers in lymphatics in COPD patients. These studies indicate that the lung LECs undergo inflammatory changes associated with cigarette smoke exposure and increased thrombin in COPD. The results of this study reinforce the evidence that lymphatic dysfunction occurs early in the pathogenesis of COPD caused by cigarette smoke, which is associated with increased thrombin and fibrin clots in lung lymph.

Primary lymphatic anomalies and immune homeostasis (Pearce et al.). Pearce et al. investigate how primary lymphatic anomalies (PLA), a group of developmental disorders affecting lymphatic vessels, affect immune homeostasis. Examination of both retrospective data from 177 PLA patients and a prospective cohort of 28 patients alongside 20 healthy controls, remarkably uncovers a selective reduction in circulating lymphocytes, which preferentially depletes naive CD4^+^ T cells. Interestingly, genital oedema and the likelihood of concomitant intestinal lymphangiectasia independently predicted CD4^+^ T cell depletion. The findings in this study underscore the role of lymphatic–lymphocyte interactions in immune regulation and increased circulating Tregs.

These articles highlight the significance of lymphatic dysfunction and anomalies in various pathological conditions.

## Therapeutic and engineering approaches

Therapeutic approaches to central nervous system (CNS) diseases via the meningeal lymphatics (Zhang et al.). This review article outlines the roles of meningeal lymphatic vessels and the glymphatic system in maintaining fluid balance, clearance of neurotoxic proteins, and immune surveillance in the brain. Importantly, dysfunction in either pathway has been linked to various CNS diseases such as Alzheimer’s disease, Parkinson’s disease, and multiple sclerosis. This article explores therapeutic strategies targeting the meningeal lymphatic system for delaying or preventing neurological diseases, including the administration of VEGF-C. Therapeutic drug delivery mediated by the meningeal lymphatic system can offer a novel approach for the treatment of CNS diseases, potentially bypassing the blood-brain barrier.

Biomaterial-based lymphedema therapy (Deshpande et al.). This review article provides the current understanding of lymphatic tissue engineering with a focus on scaffold materials, lymphangiogenic factors, and regenerative strategies to recapitulate lymphatic vessels and nodes. It also addresses current challenges in clinical translation to treat lymphedema and other lymphatic disorders. Ongoing research efforts include optimization of scaffold composition and mechanical properties, as well as scalable production, to enhance therapeutic efficacy.

These articles shed new light on potential therapeutic strategies for CNS diseases and lymphedema. While clinical translation will require addressing biological and technical barriers, ongoing research holds great promise for the development of effective therapies for lymphatic disorders.

In conclusion, the articles on this Research Topic showcase exciting advances in our understanding of the organ-specific functions of the lymphatic system, spanning from developmental signaling pathways to tissue repair and disease processes. They also underscore the vast diversity and clinical importance of lymphatic dysfunctions and therapeutic innovation. However, despite these advances, critical gaps persist in understanding how tissue microenvironments dictate organotypic lymphatic function.

Overall, this Research Topic highlights the importance of the lymphatic system in tissue homeostasis in health and disease. It also emphasizes the need for further understanding of the molecular and cellular heterogeneity in the lymphatic system. We anticipate that this Research Topic will inspire integrative studies and therapeutic development to harness the lymphatics in disease modulation.
